# Draft Genome Sequence of Listeria monocytogenes LM201, Isolated from Foodstuff

**DOI:** 10.1128/genomeA.01417-14

**Published:** 2015-02-05

**Authors:** Yongpeng Wu, Jinshui Zheng, Yunxiao Wang, Shaowen Li, Hui Jin, Zili Li, Dingren Bi, Ming Sun, Mei Liu

**Affiliations:** a Laboratory of Veterinary Microbiology and Immunology, College of Veterinary Medicine, Huazhong Agricultural University, Wuhan, People’s Republic of China; b State Key Laboratory of Agricultural Microbiology, Huazhong Agricultural University, Wuhan, People’s Republic of China; c Key Laboratory of Development of Veterinary Diagnostic Products, Ministry of Agriculture, Wuhan, People’s Republic of China

## Abstract

Listeria monocytogenes is a facultative intracellular foodborne pathogen that can cause listeriosis in humans and animals. L. monocytogenes LM201 was isolated from foodstuff. The draft genome sequence of strain LM201 provides the genetic basis for the application of this strain in biotechnological vaccine production.

## GENOME ANNOUNCEMENT

Listeria monocytogenes is a Gram-positive, nonsporulating, aerobic and facultative anaerobic, rod-shaped, and facultative intracellular bacterium (1, 2). It is a foodborne pathogen (3) that can cause listeriosis (gastroenteritis, meningitis, encephalitis, septicemia, and abortion) in animals and humans, especially immunocompromised individuals (the old, neonates, and pregnant women), with a mean mortality rate of 20–30% or higher (4). L. monocytogenes LM201, a serovar 4b strain, was isolated from foodstuff in Hubei province (China). The LD_50_ of strain LM201 was 6.3 × 10^6^ CFU for Kunming mice after i.p. infection. Strain LM201 was also assayed for other phenotypic characteristics and was determined to be a good candidate for developing a vaccine. Here we report the genome sequence of L. monocytogenes LM201, which provides the genetic basis for rationally designing and generating defined virulence-attenuated strains for biotechnological vaccine production.

Genomic DNA of the strain LM201 was sequenced with a whole-genome shotgun strategy using an Illumina HiSeq 2000 platform. Two different sequencing libraries (300-bp paired end and 5,000-bp mate pair) were prepared. In total, 43,930,390 paired-end reads of 100 bp and 20,921,890 mate-pair reads of 90 bp were obtained from the sequencing (1,910-fold genome coverage). *De novo* assembly was done using ABySS version 1.3.4 software (5), yielding 86 contigs with an *N*_50_ size of 226,587 bp and a maximum contig size of 342,356 bp. A total of 66 scaffolds were produced, with a total size of 3,200,729 bp and a G+C content of 37.44%.

Genome annotation was accomplished using the RAST server (6). Gene prediction revealed 3,165 protein-coding genes (CDSs), 6 rRNA operons, and 35 tRNA genes. Among the protein-coding genes, there were 2,736 genes with function prediction; 2,504 were identified in the COG database; and 2,674 were identified in the Pfam database.

### Nucleotide sequence accession number.

The L. monocytogenes LM201 draft genome sequence has been deposited in the GenBank database under the accession number AYPT00000000. The version described in this paper is the first version.

## References

[1] Seeliger HPR, Jones D 1986 Genus *Listeria*, p 1235–1245. *In* Sneath PHA, Mair NS, Sharpe ME, Holt JG (ed), Bergey’s manual of systematic bacteriology, vol 2 Williams and Wilkins, Baltimore, MD.

[2] Freitag NE, Port GC, Miner MD 2009 *Listeria monocytogenes*—from saprophyte to intracellular pathogen. Nat Rev Microbiol 7:623–628. doi:10.1038/nrmicro2171. 19648949 PMC2813567

[3] Farber JM, Peterkin PI 1991 *Listeria monocytogenes*, a foodborne pathogen. Microbiol Rev 55:476–511. 1943998 10.1128/mr.55.3.476-511.1991 PMC372831

[4] Vázquez-Boland JA, Kuhn M, Berche P, Chakraborty T, Domínguez-Bernal G, Goebel W, González-Zorn B, Wehland J, Kreft J 2001 *Listeria* pathogenesis and molecular virulence determinants. Clin Microbiol Rev 14:584–640. doi:10.1128/CMR.14.3.584-640.2001. 11432815 PMC88991

[5] Simpson JT, Wong K, Jackman SD, Schein JE, Jones SJM, Birol İ 2009 ABySS: a parallel assembler for short read sequence data. Genome Res 19:1117–1123. doi:10.1101/gr.089532.108. 19251739 PMC2694472

[6] Aziz RK, Bartels D, Best AA, DeJongh M, Disz T, Edwards RA, Formsma K, Gerdes S, Glass EM, Kubal M, Meyer F, Olsen GJ, Olson R, Osterman AL, Overbeek RA, McNeil LK, Paarmann D, Paczian T, Parrello B, Pusch GD, Reich C, Stevens R, Vassieva O, Vonstein V, Wilke A, Zagnitko O 2008 The RAST server: Rapid Annotations using Subsystems Technology. BMC Genomics 9:75. doi:10.1186/1471-2164-9-75. 18261238 PMC2265698

